# Regionally Divergent Relationships Between Microbial Communities and Environmental Gradients in Potato Production Systems

**DOI:** 10.1111/1758-2229.70437

**Published:** 2026-10-20

**Authors:** Shan Shan, Isabelle George, Linda L. Kinkel, Richard A. Lankau

**Affiliations:** ^1^ Department of Plant Pathology University of Wisconsin‐Madison Madison Wisconsin USA; ^2^ Department of Plant Pathology University of Minnesota St. Paul Minnesota USA

**Keywords:** crop rotation, potato, soil health, soil microbial community

## Abstract

Using soil microbiome data as indicators of soil health remains a challenging task in agriculture research. Microbiome data are complex and exhibit strong spatiotemporal variation and diverse responses to environmental factors. As a result, measurements of microbiota often are difficult to interpret and do not provide credible inferences about soil health status. In this study, we evaluated the distributions of soil microbial communities in three distinct potato growing regions of the Upper Midwest of the USA and compared their relationships with soil chemistry and management factors across regions. A total of 1270 soil samples were collected and analysed for microbial properties, including bacterial and fungal abundance, taxonomic and functional diversity and composition. We found substantial variation in the microbial community properties across spatial scales. Environmental factors were more strongly associated with community diversity at the class level than at finer taxonomic levels. Soil pH, soil organic matter (SOM), crop rotation schemes and fumigation correlated with different aspects of microbial properties, while most of the relationships were region‐specific. Our results suggest that the microbiological component of soil health includes multiple aspects of the community properties, and region‐specific recommendations are needed for developing soil health promoting practices.

## Graphical abstract

In the potato‐growing systems of the Upper Midwest of the US, soil pH, organic matter (SOM), rotation length (RL), and rotation crop diversity (RD) had varying effects on soil microbial abundance and taxonomic diversity, depending on the specific growing region.

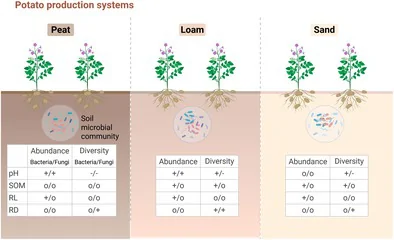

## 1 Introduction

Soil microorganisms are an important component of soil health. Soil health includes physical, chemical, and biological attributes, and numerous indicators have been developed to assess soil health based on their relationship with management factors (Raghavendra et al. 2020). The biological components of soil health have always been more difficult to access due to the difficulty in establishing appropriate indicators to reflect microbial‐mediated multi‐dimensional ecosystem functions (Mahdi et al. 2017; Schloter et al. 2018). Current biological indicators include macrofauna, microbial nutrients, and microbial processes such as the transformation of nutrients (USDA NRCS, n.d.). Despite being the centre of microbially mediated soil processes, using microbial community composition data as a soil health indicator or making credible inferences about soil health is less straightforward (Fierer et al. 2021) due to the strong geographic differences in the community structure and complicated structure–function relationships. Using microbiome data as biological indicators may need to be geographic‐specific and require a comprehensive understanding of their spatial distributions and relationships with environmental factors.

Soil microbial communities exhibit significant variation across different spatial scales. Many studies have investigated the spatial distribution of microbial communities across regional, continental, and global scales (Griffiths et al. 2011; Delgado‐Baquerizo et al. 2018; Wu et al. 2024). However, our understanding of the magnitude of variation in microbial abundance, community diversity, and composition at different spatial scales in agricultural soils is still limited (Neupane et al. 2022). Microbiota may exhibit the highest level of spatial heterogeneity at small spatial scales (Hugoni et al. 2021) for two reasons: (1) high niche diversity can arise at this scale from substantial variability in the soil physical and chemical properties (Mzuku et al. 2005); (2) diverse responses of individual populations to spatial heterogeneity can further complicate microbial distribution, leading to highly structured patterns even within relatively homogeneous habitats (Franklin and Mills 2003). For instance, Ramette and Tiedje (2007) found that variation in the community diversity and composition within the bacterial genus *Burkholderia* was greater in the root zone than across an agricultural field. Nevertheless, the relative contribution of spatial factors at varying scales to microbial variation likely depends on the specific soil system and the spatial factor being investigated.

Microbial community data is deep, with information preserved at each taxonomic level. Description of microbial community has mostly focused on single taxonomic levels in past literature. However, there are many questions regarding how far we need to dissect the immense amount of information down to the higher taxonomic levels for understanding their distribution and functions (Philippot et al. 2010). Soil chemistry and agricultural management are expected to influence microbial community structure at both narrow (species, genus) and broad (phylum, class) taxonomic levels (Philippot et al. 2010; Ibarbalz et al. 2013); however, the extent of their contribution might differ. Biogeographic patterns in soil microbes are thought to result from both environmental heterogeneity and other less‐deterministic processes such as drift and dispersal limitation (Griffiths et al. 2011; Chytrý et al. 2012). Niche conservatism suggests that phylogenetically related clades tend to have similar physiological adaptations to environmental constraints (Wiens et al. 2010). If adaptations to different environmental niches have shaped bacterial lineages during evolution, these environmental signals should be reflected at broader taxonomic resolution. This suggests that a strong link might exist between environmental factors and broad taxonomic community structure. On the other hand, individual taxa within a clade selected for an ecological niche might be additionally influenced by a collection of other mechanisms, including (1) selection through negative and positive species‐species interactions, (2) drift caused by random events in birth, death and dispersal, and (3) diversification through rapid evolution at fine genetic resolution (Nemergut et al. 2013; Gao et al. 2020; Barbour and Martiny 2024). These mechanisms would contribute to a weaker association between environmental factors and the community structure at fine taxonomic levels.

Factors mediating soil microbiota often change depending on the geographic context (Van Horn et al. 2013). Scale is particularly important when making inferences about ecological relationships and developing management strategies (Franklin and Mills 2009). Precision agriculture requires understanding the scale at which a parameter matters for generating management decisions that will result in a response in yield and production. Therefore, multi‐scale analyses across various geographic contexts are crucial for soil microbiome research. The potato production systems of the Upper Midwest span across three distinct regions. Soil chemistry and management practices, including crop rotation and fungicide application, varied across and within regions, providing an excellent opportunity to investigate the spatial variability of soil microbiota and the environment‐microbial relationships across spatial scales.

The goal of this study was to evaluate the spatial distribution of soil microbial communities in the Upper Midwest potato growing systems, compare drivers of microbial distribution across taxonomic levels, and compare determinants of microbial abundance and diversity across and within growing regions. We aimed to answer three questions: (1) At which spatial scales, from regions to farms to fields, will soil microbial abundance, taxonomic profiles, and functional profiles vary the most? (2) How do season, spatial location, soil chemistry and management contribute to variation in microbial communities across taxonomic scales? (3) Do edaphic and management factors have consistent or region‐specific effects on the growing‐season soil microbial abundance and diversity? We hypothesized that: (1) microbial community would show the highest variation at the field scale; (2) soil and management factors would explain more of the microbial variation at the broad taxonomic levels than at the narrow taxonomic levels; and (3) environmental factors would have different impacts on microbial communities across growing regions.

To elucidate soil microbiota distributions across diverse potato growing regions of the Upper Midwest, we collected 1270 soil samples from three potato growing regions and analysed soil chemistry, microbial abundance, taxonomic diversity and composition, and functional diversity and composition. We characterized bacterial and fungal communities and examined the multi‐scale spatial distribution of their structures, along with the soil and management factors influencing these spatial patterns.

## 2 Materials and Methods

### 2.1 Site Description and Soil Collection

Minnesota (MN) and Wisconsin (WI) are two major potato‐growing states in the Upper Midwest of U.S. Potatoes are grown in a variety of soils, including the peat soils of northern MN, the loamy soils of northern WI, and the sandy soils found in central WI and southeast MN. The peat region includes north MN, with drained peatland for specialty crops. The soils were histosols (Hemists) formed in swamps and bogs of glacial lake plains (Anderson and Grigal 1984). The loam region includes northern WI Langlade and Vilas counties, with a productive agriculture soil Antigo silt loam (Haplic Glossudalfs), which is an organic enriched and well‐drained soil with 50–100 cm of loess or silty alluvium overlie sand and gravel (Bockheim and Hartemink 2017). The sand region includes central WI sand plains, with deep and excessively drained sandy soils formed on Late Cambrian sandstone. The sand region also includes central MN, with sandy soils of varying thickness formed from glacial outwash. Our study sites were 218 commercial potato fields located in these three regions.

Soil was collected between November 2018 and July 2021, spanning three growing seasons (2018–2019, 2019–2020, 2020–2021). Two to three sampling locations (at least 100 m apart from each other) were randomly selected within each commercial field. At each sampling location, soil was collected twice: once in late fall (November) and again the following summer (July). This resulted in a pre‐plant and a growing‐season soil sample for each location. We sampled the top 15 cm soil by extracting 3–4 soil cores and mixing them in a Ziploc bag. A total of 1270 soils from 218 fields were sampled, with 530 soils (94 fields), 420 soils (74 fields), and 320 soils (50 fields) collected in 2018–2019, 2019–2020, and 2020–2021, respectively. Those fields were owned by 24 farms (ranging from 1 to 27 fields per farm) and were distributed across the three regions: 40 fields in peat, 40 fields in loam, and 138 fields in the sand region.

### 2.2 Soil Chemistry

Soil collected in the fall of each growing season was air‐dried and measured for a variety of physicochemical properties in the A&L Great Lakes Lab (Fort Wayne, IN). Soil pH was measured in a 1:1 soil/water mixture. Soil organic matter (SOM) content was quantified with loss‐on‐ignition. Concentrations of soil phosphorus (P), potassium (K), calcium (Ca), and magnesium (Mg) were measured by extracting the soil with Mehlich3 and subsequently analysed with Inductive Coupled Plasma‐Atomic Emission Spectroscopy (ICP‐AES). Soil cation exchange capacity (CEC) was calculated with the ammonium acetate equivalent values for the total cations measured in ICP, reported as milliequivalents per 100 g of soil.

### 2.3 Management History

The crop rotation history and pre‐plant fumigation information for each field were collected from the growers. We summarized the crop rotation history into two indices, rotation length and rotation diversity. Rotation length measures the number of years since the last time this field was planted with potato. For fields with no record of potato production in the past 10 years, we set rotation length equal to 10. Rotation diversity measures the number of different crop species present in the 3 years prior to the current potato year, including rotation crops and cover crops. We also recorded whether each field was fumigated pre‐planting for the year of soil sampling. In this system, soils were primarily fumigated with metam sodium, while chloropicrin was used only occasionally. Fumigation typically occurred in the fall (early November) prior to the potato growing season. Metam sodium and chloropicrin were applied by surface injection to a depth of 8–10 inch at rates of 40 gal/acre and 100 lb/acre, respectively.

### 2.4 Soil Microbial Community

#### 2.4.1 Microbial Abundance

Soil DNA was extracted from 0.25 g of frozen field soils using the DNeasy PowerSoil Pro DNA isolation kit (Qiagen, CA) following the manufacturer's instructions. Soil bacteria and fungi abundance were measured using quantitative PCR targeting bacterial 16S rRNA and fungal ITS, with primer sets Eub338 (ACTCCTACGGGAGGCAGCAG) and Eub518 (ATTACCGCGGCTGCTGG) for total bacteria (Fierer et al. 2005), and ITS86F (GTGAATCATCGAATCTTTGAA) and ITS4 (TCCTCCGCTTATTGATATGC) for total fungi (Op De Beeck et al. 2014). We created standard curves for each PCR using 10‐fold serial dilution of the target gene cloned into an *E. coli* plasmid (pGem‐T Vector, Promega, Madison, WI). Standard curves showed PCR amplification efficiencies of 90%–105%, with *r* ^2^ > 0.99. Each qPCR reaction contained 5 μL of Sooadvanced Sybr Green supermix (Bio‐rad, Hercules, CA), 0.4 μL of each of the 20 μM forward and reverse primers, 2.7 μL nuclease free water, and 1.5 μL diluted soil DNA. PCR cycling conditions were 95°C for 5 min; 38 cycles of 95°C for 15 s, 47°C (bacteria, 55°C for fungi) for 30 s, and 72°C for 30 s; and a melting curve from 65°C to 95°C with increase of 0.5°C per cycle. The optimal annealing temperatures 47°C and 55°C were determined using gradient PCR.

#### 2.4.2 Taxonomic Profile

Soil microbial communities were characterized by high‐throughput sequencing of the bacterial 16S rRNA and fungal ITS2 gene regions. Extracted soil DNA was quantified on a NanoDrop and then used for constructing 16S and ITS amplicon libraries in the University of Minnesota Genomics Center. The DNA template was standardized and amplified with the primer sets V3F (CCTACGGGAGGCAGCAG) and 806R (GGACTACHVGGGTWTCTAAT) for the V3‐V4 region of bacterial 16S rRNA, and 5.8S (TCGATGAAGAACGCAGCG) and ITS4 (TCCTCCGCTTATTGATATGC) for the eukaryotic ITS2 region (Wang et al. 2015; Gohl et al. 2016). The final PCR products were normalized, pooled, and cleaned up before sequencing in a pair‐end 2 × 300 bp run on an Illumina MiSeq platform at the University of Minnesota Genomics Center.

Sequencing data were processed using Qiime 2. We first used Cutadapt to remove the forward and reverse primers of the ITS reads. Trimmed ITS reads and the untrimmed 16S reads were truncated to allow a minimum overlap of 20 nucleotides, quality filtered to a maxEE of four, denoised, paired‐end merged, and chimeras removed using the DADA2 pipeline (Shan et al. 2024). Taxonomy was assigned to the feature table of amplicon sequence variant (ASV) using Qiime2's feature‐classifier plugin, using the RDP Naïve Bayesian Classifier fit to the SILVA 138 database for 16S reads and UNITE database for ITS reads. Mitochondrial and chloroplast sequences were excluded using the feature‐table plugin.

The bacterial ASV table included 352,862 unique ASVs for 1270 samples. The fungal ASV table was first filtered by removing ASVs that do not belong to Fungi Kingdom, leaving a total of 36,374 fungal ASVs for 1176 samples. Bacterial and fungal ASV tables were then merged at the class, order, family, and genus levels using the package phyloseq and the “tax_glom” function in R (version 4.1.2). Merging produced 218 classes (96.9% of the 352,862 ASVs identified to a class), 555 orders (95.6%), 958 families (93.7%), and 2245 genera (86%) for bacteria; 81 classes (90.3%), 229 orders (87.3%), 611 families (82.2%), and 1755 genera (77.4%) for fungi. Alpha diversity was measured as the Inverse Simpson index after rarefying samples to the sample depths that retained as many samples as possible (1800 reads for bacteria, 700 reads for fungi, samples under these thresholds were omitted, Figure S1A,B) to account for variation in sequencing depth using the vegan package. Compositional variation of bacterial and fungal communities was visualized using Principal Coordinate Analysis (PCoA) based on Bray–Curtis dissimilarity with unrarefied count data transformed to relative abundance, which preserves statistical power while accounting for compositionality. Permutation multivariate analysis of variance (PERMANOVA) was performed to compare microbial community composition among regions and seasons using the “adonis” function in the vegan package. Two‐way analysis of variance was used to compare microbial abundance and diversity between regions and seasons.

#### 2.4.3 Functional Profile

We used PICRUSt2 (v2.6.0) to predict the metabolic functions of the 16S and ITS community. Following the standard PICRUSt2 workflow (Douglas et al. 2020), the 16S ASV table was matched to the PICRUSt2‐SC database (Wright and Langille 2025) using the default maximum Nearest Sequenced Taxon Indices (NSTI) cutoff of 2.0 to predict functional gene content, including Enzyme Commission (EC) numbers, KEGG orthologs (KO), and MetaCyc pathway abundances. Abundance tables were then normalized by the predicted 16S gene copy number and the abundance of the representative sequences to obtain the predicted abundance per sample. Of the 352,862 sequences, 350,705 aligned to the reference sequences, of which 350,192 passed the NSTI cutoff and were used for prediction. The ITS ASV table was analysed using a similar workflow and matched to the ITS database. The ITS sequences poorly aligned to the database, with only 15,619 out of 108,420 sequences passing the alignment threshold (min_align = 0.8) and used for functional prediction. A final table of EC numbers per sample was obtained for ITS prediction. In this study, we interpreted the ITS‐based results with caution because of this limitation associated with fungi prediction.

We exported the predicted 16S KO and pathway abundance tables and ITS EC table into R to analyse functional diversity and composition for 16S and ITS community, respectively. Functional diversity was measured using the Inverse Simpson index in the Vegan package, and functional composition was visualized using PCoA as described above.

### 2.5 Data Analysis

(1) *At which spatial scales, from regions to farms to fields, will soil microbial abundance, taxonomic profiles, and functional profiles vary the most?* We decomposed the effect of spatial location into three scales: region, farm, and field using linear mixed effect models. The models included region, farm, and field as random factors, with farm nested within region and field nested within farm. Season was also included in the model as a fixed term. R function “decompR2” from the decompR2 package was used to decompose the total variance into the variance of the fixed effects, random intercepts, and residuals (Matre 2022). Decomposed variance was calculated as the mean increase in R^2^ when a factor was added into the model. Response variables for this analysis included abundance, taxonomic diversity at five phylogenetic levels (class, order, family, genus, and ASV), and functional diversity. For the community composition, Permutation Multivariate Analysis of Variance (PERMANOVA) was used to decompose the variance among the three spatial scales. To account for the hierarchical structure of the three spatial location factors during variance partitioning, we fitted region as the first term in the model, followed by farm and field, with season as the last term in the model.

(2) *How do season, spatial location, soil chemistry and management contribute to variation in microbial communities across taxonomic scales?* We used variance partitioning analysis to disentangle the variance in the microbial community explained by four components: season (fall and summer), spatial location (region, farm, and field), soil chemistry (pH, SOM, CEC, P, K, Mg, and Ca), and management (rotation diversity and length). Fumigation was not included in this analysis, because pre‐plant soils were collected around the time of fumigation, either before or immediately after fumigation, and thus fumigation effects would not yet be evident in DNA‐based measurements. R function “varpart” from the vegan package was used to calculate variance explained by each of the four factors, portions of variance jointly explained by multiple factors, and portions of variance by each factor when modeled individually. All numerical variables were standardized using the “scale” function to remove the unit differences. The partitioning results were visualized with Upset plot. This analysis was performed for bacterial and fungal abundance, taxonomic diversity, taxonomic composition at various phylogenetic levels, as well as functional diversity and composition of all soil samples. For abundance and diversity, the total variation in the response variables was partitioned by linear regression; for the community composition, the total variation in the community represented by Bray‐Curtis distance matrix was partitioned based on distance‐based redundancy analysis.

(3) *Do edaphic and management factors have consistent or region‐specific effects on the growing‐season soil microbial abundance and diversity?* We used linear regression to investigate the effect of soil chemistry (soil pH, OM, P, K), rotation history (rotation diversity and length), and fumigation (whether fumigated or not before planting) on growing‐season soil microbial properties across and within growing regions. Response variables for this analysis included the abundance, class‐level taxonomic diversity, and functional diversity of bacterial and fungal communities measured in the summer. Because variation partitioning produced similar results for bacterial functional diversity based on the KO and pathway abundance tables, we included only the KO table‐based functional diversity for bacteria in this analysis. We selected taxonomic diversity at the class level because edaphic and management factors explained more variance at this taxonomic level than at any other level. Fall communities were not included for this analysis because fall soils were collected either before or shortly after fumigation, making them unsuitable for this analysis. We focused on four soil factors of interest—pH, SOM, P, and K—as there was minimum multicollinearity among these variables as assessed by variance inflation factors (VIF). A linear mixed effect model was constructed for each microbial property with soil chemistry, rotation history, fumigation, and their interactions with region as the fixed effects, as well as field and farm as random effects to account for the nested structure of the sampling. Region was a categorical variable including three levels: peat, loam, and sand. We evaluated multicollinearity among pH, SOM, P, K, rotation history, and fumigation using VIF and found no significant collinearity issues. Main and interaction effects were subsequently evaluated using a Type III ANOVA on the mixed effect model. A significant interaction between region and one of the soil chemistry or management factors indicates a non‐consistent effect of this factor across the three growing regions. We then visualized the correlations between these environmental factors and microbial abundance and diversity for each of the three regions in scatter plots.

## 3 Results

### 3.1 Descriptive Statistics for Soil Microbial Properties and Soil Chemistry

The three potato growing regions showed distinct soil chemical properties (Table 1). Soil pH was highest (mean, 7.2) in the peat region, lower in the sand (6.1), and lowest in the loam region (5.7). The average SOM content was 58.3% in the peat region, significantly higher than the loam (2.8%) and sand regions (2.2%). Loam soils had higher mean P and K content than peat and sand regions, whereas peat soils had higher mean CEC, Ca and Mg level than loam and sand soils.

**TABLE 1:** Soil chemistry of the three potato growing regions.

	Peat	Loam	Sand
pH	7.2 (0.3)^a^	5.7 (0.6)^c^	6.1 (0.7)^b^
SOM (%)	58.3 (12.8)^a^	2.8 (0.7)^b^	1.6 (1.5)^b^
P (ppm)	19.5 (8.7)^b^	203.4 (84.8)^a^	197.1 (112.3)^a^
K (ppm)	125.7 (58.1)^c^	250.6 (156.4)^a^	165.7 (118.7)^b^
Ca (ppm)	5811.7 (3768.8)^a^	882.2 (394.6)^b^	728.1 (381.9)^b^
Mg (ppm)	708.3 (111.3)^a^	112.4 (47)^b^	120.9 (68.8)^b^
CEC (meq/100 g)	34.8 (13.9)^a^	8.2 (2.6)^b^	6.6 (2.8)^c^

*Note:* Values show the mean and standard deviation in parenthesis. Different superscript letters indicate significant differences among groups (ANOVA followed by Tukey's test, *p* < 0.05).

Abbreviation: SOM, soil organic matter.

Soil microbial abundance, taxonomic and functional diversity differed among three regions and between seasons (region, region×season, *p* < 0.05, Figure 1). Bacterial abundance and class‐level taxonomic diversity followed the pattern peat > loam > sand, whereas bacterial functional diversity showed the reverse trend (sand > loam > peat). Fungal abundance and functional diversity followed this pattern peat = loam > sand, whereas class‐level taxonomic diversity was lowest in peat. Genus‐level bacterial and fungal taxonomic diversity showed minimum differences among regions. Summer (mid‐season) microbial abundance, taxonomic diversity, and functional diversity were generally lower than those observed in the fall (pre‐plant). Exceptions included sand soil microbial abundance, peat soil fungal taxonomic diversity, and loam and sand soil microbial functional diversity, where differences between seasons were minimal or showed the opposite trend (Figure 1).

**FIGURE 1:**
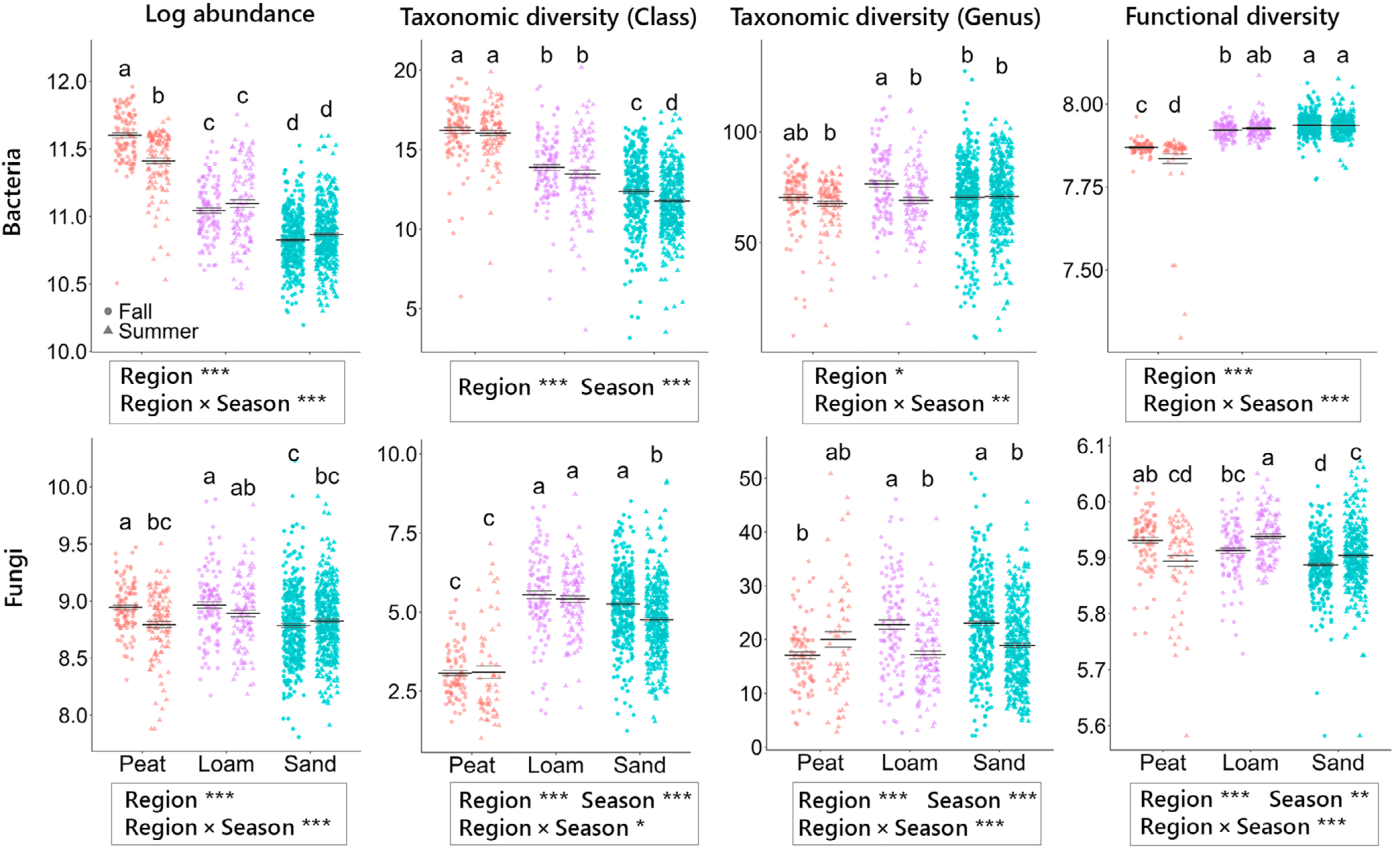
The abundance, taxonomic diversity at the class and genus level, and functional diversity of soil bacteria and fungi in the fall and summer in the three potato growing regions of upper Midwest. Functional profile was inferred from KO abundance for bacteria and EC abundance for fungi. The effect of season, region, and their interaction were evaluated using two‐way analysis of variance. Different letters indicate significant differences based on Tukey's post hoc test. ***, *p* < 0.001, **, *p* < 0.01, *p < 0.1.

We visualized the microbial taxonomic and functional composition with PCoA based on the Bray‐Curtis metric (Figure 2). We found that both region and season structured the bacterial and fungal communities. Bacterial taxonomic and functional composition in the peat region soils were noticeably separated from those of loam and sand regions (region, *p* = 0.001). Fungal taxonomic composition showed clear separation among the three regions (region, *p* = 0.001), while fungal functional composition showed weaker yet significant separation (region, *p* = 0.001, Figure 2). Microbial taxonomic and functional compositions showed clear separation between fall and summer (season, *p* = 0.001, Figure S2); however, the seasonal differences were generally smaller than those associated with region (e.g., *r* ^2^ = 0.01 vs. *r* ^2^ = 0.16 for bacterial taxonomic composition).

**FIGURE 2:**
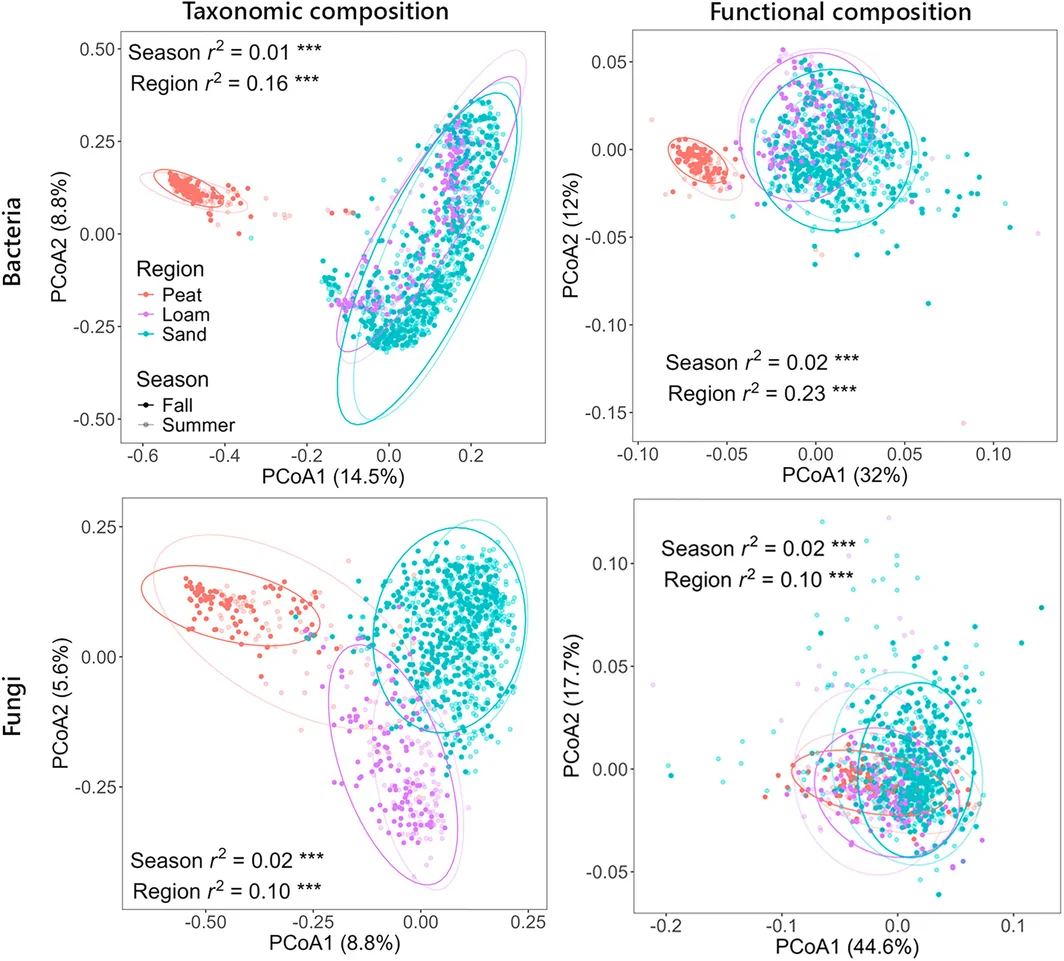
Principal coordinate analysis (PCoA) of bacterial and fungal taxonomic and functional compositions based on Bray–Curtis dissimilarity. Functional composition was inferred from KO abundance for bacteria and EC abundance for fungi. The effects of season and region were detected by permutation multivariate analysis of variance. ***, *p* < 0.001.

(1) *At which spatial scales, from regions to farms to fields, will soil microbial abundance, taxonomic profiles, and functional profiles vary the most?* Variance partitioning across the three spatial scales—region, farm, and field—generally showed that field explained most of the variance, followed by farm, while region explained the least amount of variance for bacterial and fungal abundance, bacterial and fungal taxonomic and functional diversity, as well as fungal taxonomic and functional composition (Table 2). Bacterial taxonomic and functional composition showed a different pattern, with the majority of variance explained by region, followed by field and farm. These trends were generally consistent across taxonomic scales (Table 2).

**TABLE 2:** Variance partitioning at different spatial scales for microbial properties, where the effect of spatial location was decomposed into portions explained by region, farm, and field.

	Bacteria					Fungi				
	Class	Order	Family	Genus	ASV	Class	Order	Family	Genus	ASV
Taxonomic diversity										
Region	12.6	2.9	3.2	1.1	2.0	7.6	3.9	2.0	1.2	1.3
Farm	20.2	12.6	11.2	8.6	7.6	12.9	7.1	7.7	9.2	8.7
Field	30.7	24.1	23.3	19.5	15.7	22.2	19.2	22.8	20.5	19.1
Taxonomic composition										
Region	31.7	30.9	32.0	29.2	17.2	10.8	10.1	11.8	10.7	11.4
Farm	16.4	17.7	17.7	18.9	16.3	13.6	12.5	12.9	13.9	14.6
Field	18.7	18.6	18.2	18.7	23.4	21.5	23.9	24.3	24.7	25.6

	Abundance		Functional diversity			Functional composition		
	Bacteria	Fungi	Bacteria		Fungi	Bacteria		Fungi
			Path	KO	EC	Path	KO	EC
Region	19.1	1.4	7.0	7.8	2.1	26.4	23.5	10.1
Farm	23.3	9.3	14.4	16.1	8.4	11.2	12.7	11.0
Field	30.9	24.5	32.6	31.5	20.4	22.8	23.1	22.4

*Note:* The percentage of the total variation explained is shown.

(2) *How do season, spatial location, soil chemistry and management contribute to variation in microbial communities across taxonomic scales?* Soil chemistry explained a greater proportion of the variance in microbial taxonomic diversity at the class level than at finer (order, family, genus, ASV) levels. When modelled individually, soil chemistry accounted for 41% and 32% of the marginal variance in bacterial and fungal taxonomic diversity at the class level, respectively, compared with 5% and 6% at the order level, 14% and 7% at the family level, 7% and 2% at the genus level, and 2% and 6% at the ASV level (Table 3). Combined with season, chemistry (sum of season, chem, and season+chem, Figure 3, Table S1) explained 33% and 23% of the variance in bacterial and fungal taxonomic diversity at the class level, respectively. At finer taxonomic levels, the variance explained was 7% and 13% at the order level, 15% and 12% at the family level, 7% and 8% at the genus level, and 2% and 8% at the ASV levels for bacteria and fungi, respectively. Spatial factors explained a significant proportion of variation in microbial taxonomic diversity across taxonomic scales. After accounting for season, chemistry and rotation effects, spatial factors alone explained 19% and 13% of the remaining variance in bacterial and fungal taxonomic diversity at the class level, respectively, as well as 29% and 17% at the order level, 19% and 27% at the family level, 18% and 26% at the genus level, and 19% and 21% at the ASV level (Figure 3).

**TABLE 3:** Variance partitioning showing the effect of season, spatial location, soil chemistry, and management history on microbial properties.

	Taxonomic diversity		Taxonomic composition		Abundance			
	Bacteria	Fungi	Bacteria	Fungi		Bacteria		Fungi
Class								
Season	0.6	1.3	0.9	3.5	Season	0.1		0.0
Spatial	61.2	44.2	60.2	34.6	Spatial	72.6		32.6
Chem	41.4	31.7	43.2	15.3	Chem	58.8		8.9
Manag	11.5	12.9	15.4	5.5	Manag	23.5		0.8
Order								
Season	0.0	2.0	1.2	3.6				
Spatial	35.4	32.8	60.7	35.3				
Chem	5.0	15.9	43.2	12.4	*Functional* diversity			
Manag	0.8	8.5	15.5	4.6		Bacteria		Fungi
Family						Pathway	KO	EC
Season	0.3	4.8	1.2	2.9	Season	0.0	0.0	0.8
Spatial	34.9	32.5	61.6	38.3	Spatial	52.0	52.9	28.3
Chem	14.4	7.0	44.0	13.8	Chem	33.8	33.9	6.2
Manag	2.0	4.1	15.8	5.5	Manag	11.9	11.6	0.4
Genus								
Season	0.0	3.3	1.3	2.6				
Spatial	26.0	28.6	60.1	38.7				
Chem	7.2	2.4	42.0	13.1	*Functional composition*			
Manag	1.5	1.8	14.7	5.0		Bacteria		Fungi
ASV						*Pathway*	*KO*	*EC*
Season	0.0	1.5	0.5	1.9	Season	1.2	1.5	2.1
Spatial	23.3	26.9	48.4	41.5	Spatial	52.0	50.7	31.6
Chem	1.9	5.8	23.6	13.3	Chem	31.4	30.0	10.0
Manag	1.9	1.4	8.1	4.8	Manag	8.8	8.1	2.7

*Note:* Values show marginal variance (%) explained by each factor when modelled individually using R function “varpart”.

**FIGURE 3:**
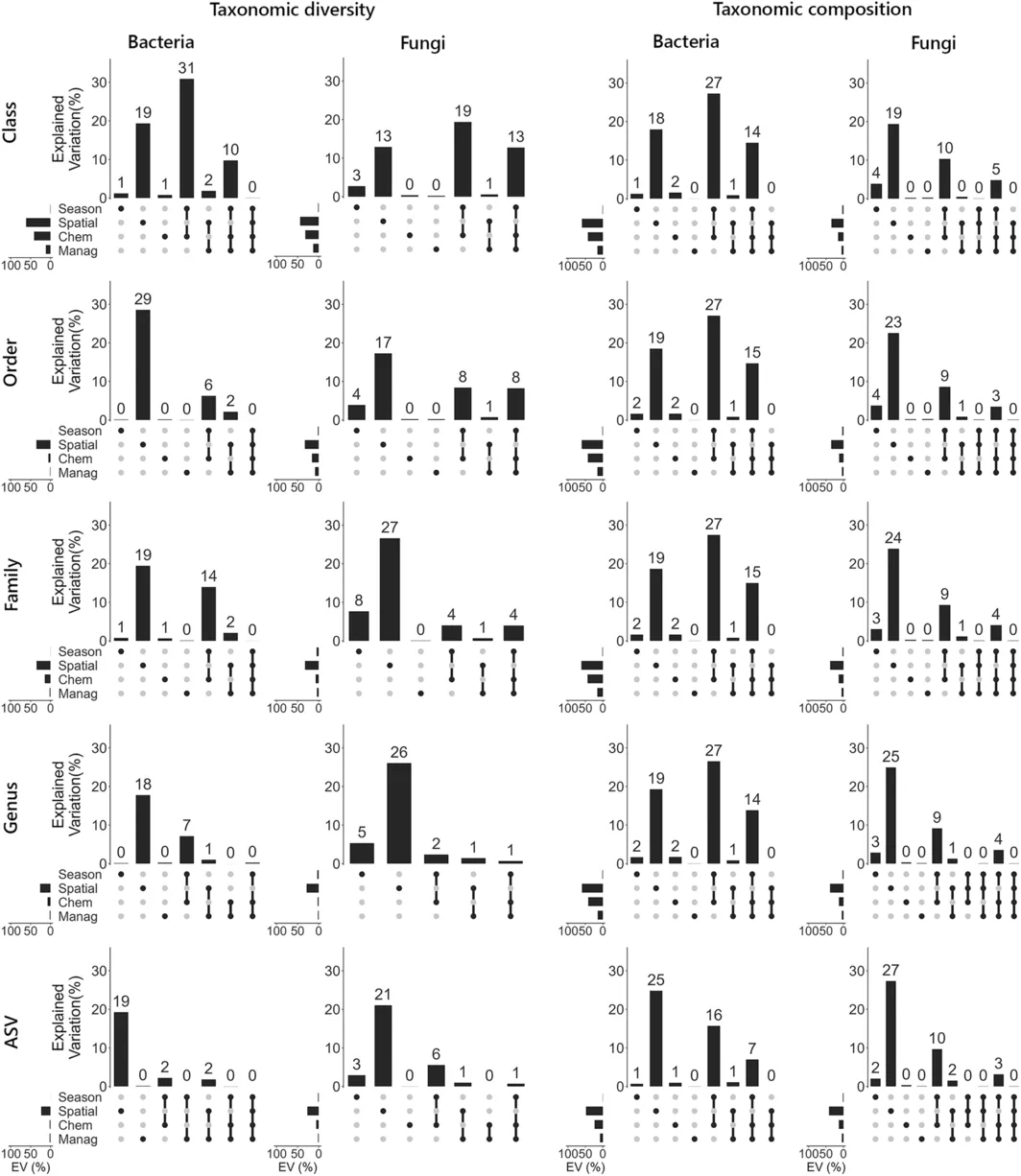
Upset plot of variance partitioning analysis showing the effect of season, spatial location, soil chemistry, and management history on microbial taxonomic diversity and composition at various phylogenetic levels. Horizontal bars show the marginal variance explained (adj. *r* ^2^) by each factor when modelled individually. Vertical bars show the unique and joint contributions of factor interactions from variance partitioning.

For taxonomic composition, soil chemistry consistently explained a significant amount of variance across taxonomic scales. When modelled individually, soil chemistry accounted for 43% and 15% of the marginal variance in bacterial and fungal taxonomic composition at the class level, respectively; 43% and 12% at the order level; 44% and 14% at the family level; 42% and 13% at the genus level; and 24% and 13% at the ASV level (Table 3). Soil chemistry and season together explained 30% and 14% of the variance at the class level, 30% and 13% at the order level, 31% and 13% at the family level, 30% and 12% at the genus level, and 17% and 12% at the ASV level for bacterial and fungal taxonomic composition, respectively (Figure 3, Table S1). Spatial factors also explained a substantial proportion of variance across taxonomic scales, independently accounting for 18% and 19% at the class level, 19% and 23% at the order level, 19% and 24% at the family level, 19% and 25% at the genus level, and 25% and 27% at the ASV level for bacterial and fungal communities, respectively (Figure 3).

Soil chemistry generally accounted for a greater proportion of variance in bacterial than in fungal properties. Together with season, it explained 38% of the variance in bacterial abundance, 23% in functional diversity, 25% in functional composition, and 30%, 30%, 31%, 30%, and 17% in taxonomic composition from class to ASV levels (Figure 3 and Figure 4, Tables S1 and S2). In comparison, soil chemistry and season explained smaller proportions of variance in fungal properties, including 9% in abundance, 7% in functional diversity, 10% in functional composition, and 14%, 13%, 13%, 12%, and 12% in taxonomic composition from class to ASV levels (Figure 3 and Figure 4, Tables S1 and S2). This pattern was less evident for taxonomic diversity. Across taxonomic scales, soil chemistry and season explained 33%, 7%, 15%, 7%, and 2% of the variance in bacterial taxonomic diversity, compared with 23%, 13%, 12%, 8%, and 8% in fungal taxonomic diversity (Figure 3, Table S1).

**FIGURE 4:**
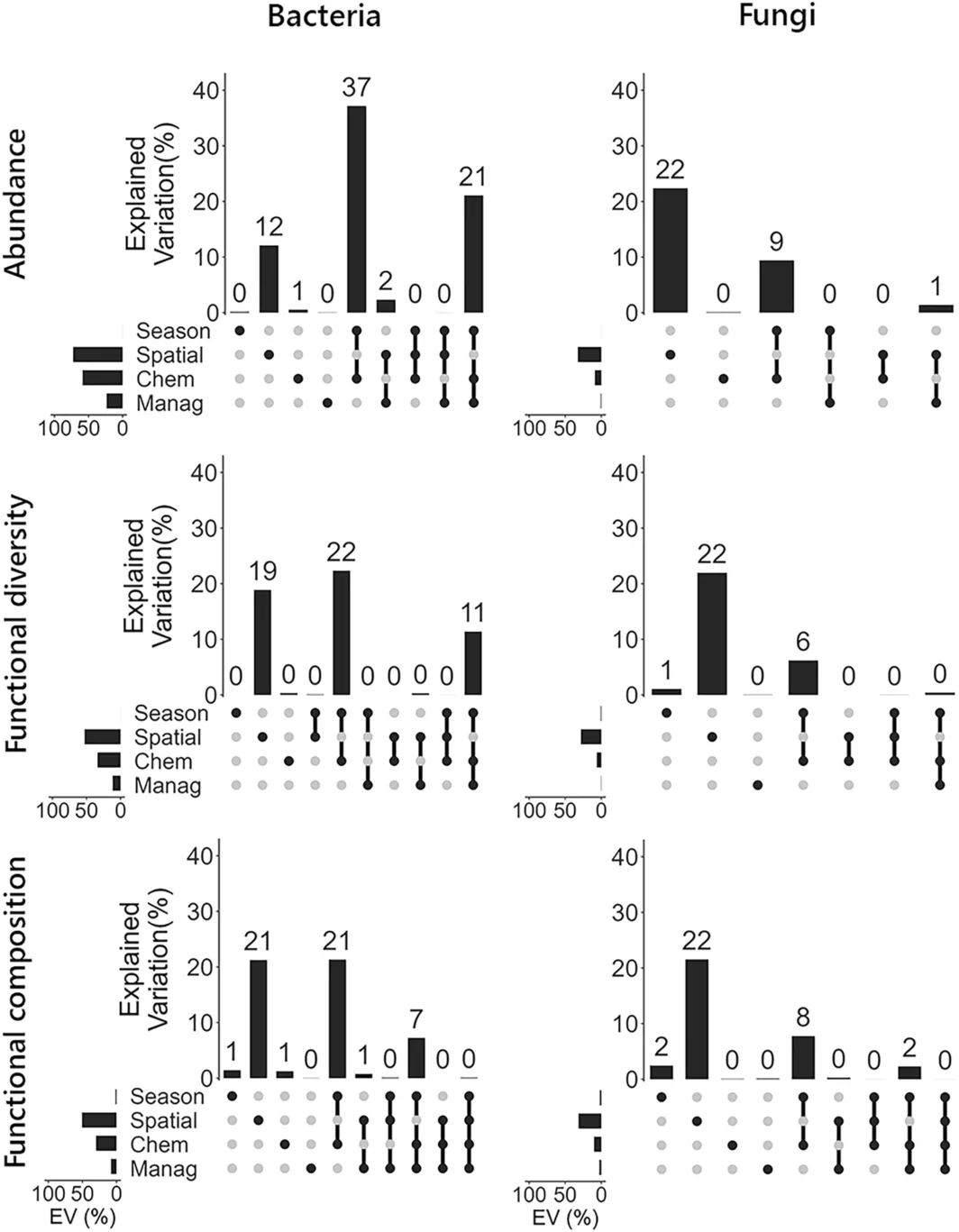
Upset plot of variance partitioning analysis showing the effect of season, spatial location, soil chemistry, and management history on microbial abundance, functional diversity, and functional composition. Functional profile was inferred from KO abundance for bacteria and EC abundance for fungi. Horizontal bars show the marginal variance explained (adj. *r* ^2^) by each factor when modelled individually. Vertical bars show the unique and joint contributions of factor interactions from variance partitioning.

Spatial factors alone generally explained a greater proportion of variance in fungal than in bacterial properties, except for taxonomic diversity at coarse taxonomic levels. After accounting for soil chemistry, season and management, space independently explained 12% of the variance in bacterial abundance, 19% in functional diversity, 21% in functional composition, and 18%, 19%, 19%, 19%, and 25% in taxonomic composition from class to ASV levels (Figures 3 and 4). In contrast, space explained 22% of fungal abundance, 22% in functional diversity, 22% in functional composition, and 19%, 23%, 24%, 25%, and 27% in taxonomic composition from class to ASV levels (Figures 3 and 4). For taxonomic diversity, space effects were stronger in bacteria than fungi at coarse taxonomic levels, explaining 19% and 29% at the class and order levels for bacteria, compared with 13% and 17% for fungi. However, this pattern reversed at finer taxonomic levels, where space explained 19%, 18%, and 19% for bacteria but 27%, 26%, and 21% for fungi at the family, genus, and ASV levels, respectively (Figure 3).

Overall, season alone explained very little of the variation in microbial communities. Season alone explained slightly higher amounts of variance in fungal taxonomic and functional diversity and composition than in bacteria. Season explained 1% of the variance in fungal functional diversity, 3% in functional composition, 3%, 4%, 8%, 5%, and 3% in taxonomic diversity, 4%, 4%, 3%, 3%, and 2% in taxonomic composition from class to ASV levels, respectively; for bacteria, season explained 0.1% of the variance in functional diversity, 1% in functional composition, 1%, 0.1%, 1%, 0.1%, and 0% in taxonomic diversity, and 1%, 2%, 2%, 2%, and 1% in taxonomic composition from class to ASV levels, respectively (Figures 3 and 4, Tables S1 and S2). Season alone contributed to < 1% variance in bacterial and fungal abundance (Figure 3, Table S2).

We found significant overlaps between space, soil chemistry, and management in explaining the variation in most microbial properties (Figures 3 and 4, Tables S1 and S2), likely due to the correlation between the three factors. For instance, soil chemistry is related to the spatial location of the sample (Table 1). Management practices are specific to each field and are thus auto‐correlated with the spatial location.

(3) *Do edaphic and management factors have consistent or region‐specific effects on the growing‐season soil microbial abundance and diversity?* Linear mixed‐effect regression showed that many of the relationships between soil chemistry and growing‐season microbial properties were region‐dependent. Soil pH influenced growing‐season soil bacterial abundance (*p* = 0.005), fungal abundance (*p* = 0.081), and fungal taxonomic diversity (*p* = 0.004), with no overall effect on bacterial taxonomic diversity (*p* = 0.981). However, all of these effects varied by region (pH × region interaction, *p* = 0.006, 0.005, 0.047, and *p* = 0.001, respectively, Table 4). An increase in soil pH was associated with an increase in bacterial and fungal abundance in loam and peat regions, but not in the sand region. Increasing soil pH was associated with higher bacterial taxonomic diversity and lower fungal taxonomic diversity in the sand and loam region, whereas in the peat region, both bacterial and fungal taxonomic diversity decreased with increasing pH (Figure 5). Soil pH had minimum impact on bacterial and fungal functional diversity (Table 4).

**TABLE 4:** ANOVA table showing the effects of soil chemistry, management, and their interactions with region on microbial abundance, taxonomic diversity at class level, and functional diversity.

	Abundance				Taxonomic diversity (class)				Functional diversity			
	Bacteria		Fungi		Bacteria		Fungi		Bacteria		Fungi	
	*F*	*P*	*F*	*P*	*F*	*P*	*F*	*P*	*F*	*P*	*F*	*P*
Soil chemistry												
pH	7.81	**0.005**	3.07	0.081+	0	0.981	8.55	**0.004**	3.10	0.079	3.24	0.073+
SOM	6.89	**0.009**	1.5	0.221	4.6	**0.032**	3.34	0.068	0.11	0.744	0.42	0.515
P	0.02	0.88	0.18	0.67	2.01	0.157	1.78	0.183	0.01	0.905	0.64	0.423
K	0.23	0.629	0.83	0.363	0.01	0.912	0.57	0.452	3.46	0.064	3.04	0.082
Management												
Rotation length	7.01	**0.009**	0.66	0.419	1.23	0.27	0.02	0.9	0.38	0.538	0.70	0.405
Rotation diversity	0.48	0.489	0	0.982	3.92	**0.049**	4.3	**0.039**	0.04	0.844	0.01	0.915
Fumigation	0.05	0.816	0.91	0.34	12.16	**0.001**	0.02	0.892	0.34	0.562	0.26	0.614
Region	2.09	0.126	4.77	**0.009**	9.45	**< 0.001**	0.89	0.413	2.36	0.098	1.34	0.265
Interactions												
Region: pH	5.25	**0.006**	5.41	**0.005**	7.37	**0.001**	3.08	**0.047**	1.61	0.202	2.18	0.115
Region: SOM	12.24	**< 0.001**	1.03	0.357	14.85	**< 0.001**	2.59	0.076	1.33	0.265	0.89	0.414
Region: P	0.09	0.916	0.14	0.868	2.28	0.104	0.81	0.445	0.04	0.962	0.49	0.614
Region: K	0.34	0.714	1.46	0.233	1.42	0.242	2.2	0.112	1.84	0.161	3.04	**0.049**
Region: Rotation length	1.92	0.151	0.39	0.678	1.79	0.171	0.59	0.555	0.29	0.748	1.14	0.322
Region: Rotation diversity	0.34	0.715	0.19	0.83	3.51	**0.033**	2.11	0.125	0.27	0.765	0.23	0.792
Region: Fumigation	0.68	0.41	0.03	0.858	7	**0.009**	2.89	0.091	0.21	0.644	0.12	0.729

*Note:* Functional diversity was inferred from KO abundance for bacteria and EC abundance for fungi. The *F* statistic represents the ratio of explained to unexplained variation, with larger *F* values indicating stronger effects of the tested factor. The '*P'* column shows the significance values (in bold).

**FIGURE 5:**
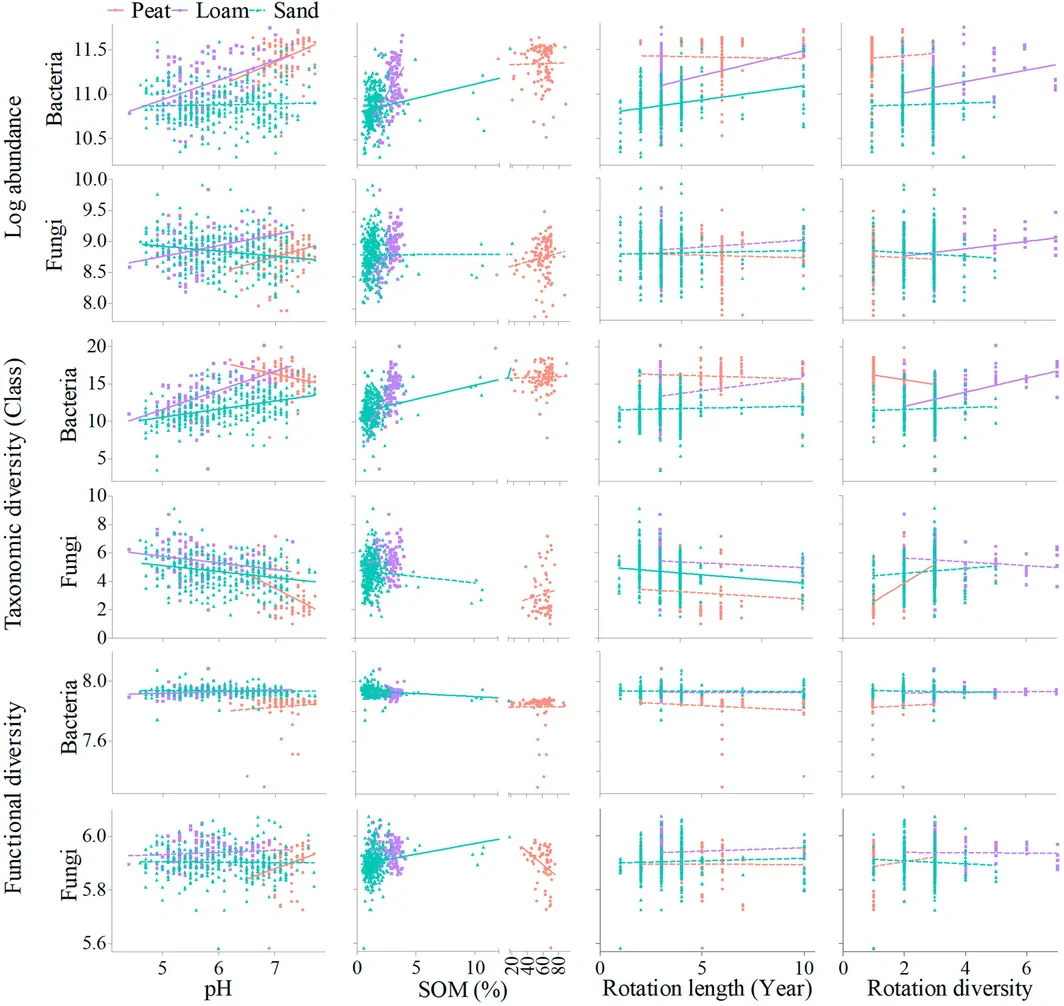
Correlation of soil and management factors with microbial abundance, taxonomic diversity at the class level, and functional diversity within each region. Functional profile was inferred from KO abundance for bacteria and EC abundance for fungi. Solid line shows significant correlation. Dashed line shows non‐significant correlation.

Soil organic matter content influenced growing‐season soil bacterial abundance (*p* = 0.009) and taxonomic diversity (*p* = 0.032) but had minimal effects on fungal abundance (*p* = 0.221), fungal taxonomic diversity (*p* = 0.068), and bacterial and fungal functional diversities (*p* = 0.744, *p* = 0.515, respectively, Table 4). Again, SOM's effect on bacterial abundance and taxonomic diversity varied across regions (SOM × region interaction, *p* < 0.001 for both): in the sand and loam regions, SOM content positively correlated with bacterial abundance and taxonomic diversity, whereas in the peat region no such relationship was observed (Figure 5). Soil P and K had no impact on microbial abundance and diversity, except for a K × region interaction affecting fungal functional diversity (*p* = 0.049, Table 4).

Rotation length influenced bacterial abundance (*p* = 0.009), and this effect was consistent across regions (region×rotation length, *p* = 0.151). Soil bacterial abundance was higher in fields with longer rotation intervals (Figure 5). Rotation length did not affect fungal abundance, or bacterial and fungal taxonomic or functional diversity (*p* = 0.419, *p* = 0.27, *p* = 0.9, *p* = 0.538, and *p* = 0.405, respectively). Rotation diversity did not affect bacterial and fungal abundance or functional diversity (*p* = 0.489, *p* = 0.982, *p* = 0.844, and *p* = 0.915, respectively), but influenced bacterial and fungal taxonomic diversity (*p* = 0.049, *p* = 0.039, respectively). Higher rotation diversity generally increased fungal taxonomic diversity but had varying effects on bacterial taxonomic diversity depending on the region (pH × region interaction, *p* = 0.033): rotation diversity increased bacterial taxonomic diversity in the loam region, but not in the sand or peat regions (Figure 5).

Fumigation mostly influenced bacterial taxonomic diversity (*p* = 0.001) and had no impact on fungal taxonomic diversity, bacterial and fungal abundance or functional diversity (*p* = 0.892, *p* = 0.816, *p* = 0.34, *p* = 0.562, and *p* = 0.614, respectively). Fumigation's effect on bacterial taxonomic diversity was also region‐dependent (pH × region interaction, *p* = 0.009): pre‐plant fumigation decreased growing‐season bacterial diversity in the loam region, but not in the sand region (Figure 6). Fumigation was not applied in the fields investigated in the peat region.

**FIGURE 6:**
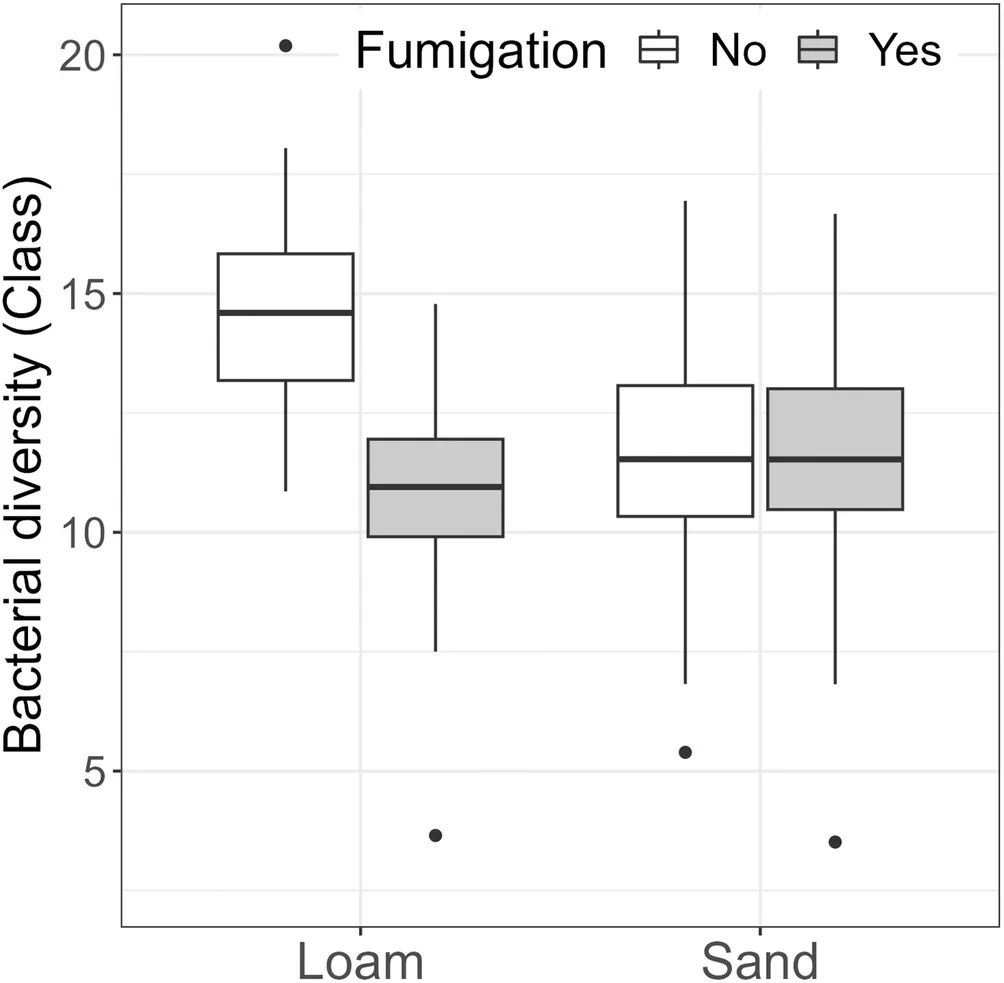
Effect of pre‐plant fumigation on mid‐season soil bacterial taxonomic diversity at the class level in the loam and sand regions.

## 4 Discussion

Here we investigated microbial communities in potato production systems across three distinct growing regions in the upper Midwest USA. Our three growing regions harboured distinct microbial communities that responded to soil chemical gradients and farm management practices in often unique ways.

(1) *At which spatial scales, from regions to farms to fields, will soil microbial abundance, taxonomic profiles, and functional profiles vary the most?* Distribution of microbial variation across different spatial scales showed that field factor was the most important source of variation for microbial abundance and diversity. This suggests that most of the differences for microbial abundance and diversity occur at small spatial scales, which is consistent with our hypothesis. It should be noted that “field” here includes both true biological variation within a field and technical variation introduced in the lab procedures. To represent the true level of variation within fields, technical replicates need to be assessed to separate the inherent variability in the sample processing and sequencing steps from consistent biological variation between sampling points in the same field. A larger sample size per field is also necessary for a more comprehensive understanding of within‐field variation. For microbial taxonomic and functional composition, while similar distribution patterns were found for fungi, bacteria followed a different pattern: region explained greater amounts of variance than farm or field. This suggests that besides the fine scale variations, bacterial composition also shows great divergence at a larger spatial scale. This is consistent with the findings in Klasek et al. (2024), where shared ASVs comprised only 14% of the bacterial sequences across nine sites in a multi‐continent potato field soil study.

(2) *How do season, spatial location, soil chemistry and management contribute to variation in microbial communities across taxonomic scales?* We found that soil chemistry explained a greater proportion of variance in microbial taxonomic diversity at the class level compared to other narrower taxonomic levels. This implies that bacterial and fungal diversities at a broad taxonomic level (class) sometimes may be sufficient for understanding their relationships with environmental factors. Change in environmental factors could influence microbial habitats, favouring or disfavoring broad groups of microorganisms with shared morphological, physiological, or life history traits that make them sensitive to those changes. This could lead to shifts in the dominant pattern of community composition, ultimately influencing the overall community diversity. For instance, increased soil temperature can enhance the selection of Bacillales within Firmicutes (Ning et al. 2020), while nitrogen deposition may favour functional groups associated with carbon and nitrogen cycling (Sun et al. 2023). These environmental changes can ultimately lead to a decrease in community diversity. However, the environmental impact may diminish as we move from broad to narrow taxonomic resolutions, as community diversity patterns become more complex due to increased functional redundancy and metabolic flexibility.

Our results suggest that environmental signals in microbial community composition may be consistent across taxonomic levels. Contrary to our hypothesis, soil chemistry explained a similar and significant amount of variation in microbial taxonomic composition across taxonomic scales. This suggests that ecological traits might be preserved to a large degree even at narrow taxonomic levels (Philippot et al. 2010). The strong link between community composition at narrow taxonomic levels and environmental heterogeneity has also been reported in Bay et al. (2020), where the turnover of soil microbial community composition across four climate zones was strongly driven by selection of local soil characteristics. Similarly, in Lu et al. (2016), the strength of the relationships between environmental variables and prokaryotic composition was comparable at the species and phylum levels in the analysis of soil and seawater samples.

Variance partitioning suggested that the relative importance of deterministic and stochastic processes in shaping contemporary community patterns may differ between bacteria and fungi. Soil chemistry significantly contributed to variation in bacterial properties, while spatial factors played a relatively larger role in influencing fungal properties. This is consistent with the hypothesis that bacterial communities are strongly influenced by deterministic processes whereas fungal communities also reflect stochastic processes (Chase and Martiny 2018). Many studies investigating ecological processes governing the assembly and maintenance of soil microbial communities show that soil bacterial community usually exhibits strong biogeographic patterns, where environmental variables strongly regulate the community composition (Bay et al. 2020); whereas the distribution of fungal community is largely dependent on dispersal limitation, and spatial distance exerted a larger effect on the community pattern (Naveed et al. 2016; Zhang et al. 2020).

Season was relatively more important in determining fungal taxonomic and functional diversity and composition than bacterial diversity and composition. Temporal control of soil fungal communities has been demonstrated in a cross‐continent study across a variety of ecosystems (Averill et al. 2019). Seasonal variability in microbial properties can be linked to weather conditions and changes in the soil environment (Rasche et al. 2011). Late fall and summer represent the most pronounced environmental differences in agricultural systems, with plant productivity peaking in the summer and root organic matter input inducing great changes in the soil community. This could exert a stronger influence on fungi than bacteria, possibly due to the close link between plant‐derived organic carbon and rhizosphere fungal communities (Cassman et al. 2016; Ai et al. 2018). Nevertheless, compared to other environmental factors, season alone explained very little variance in measured microbial properties for both bacterial and fungal communities. This is in line with observations in other agricultural fields (Zhang et al. 2020). Particularly, the resemblance between fall and summer bacterial communities suggests that the evolution of soil bacterial communities is dependent on their previous state, and samples collected in the fall could be meaningful for predicting communities in the later growing season.

We found a large proportion of microbial variation unexplained by the measured variables, consistent with many other microbial biogeographic studies (Yergeau et al. 2010; Zhang et al. 2020). This could be attributed to several unmeasured factors, including other soil physiochemical and biological properties, field management histories, climate conditions, and stochastic processes (Bay et al. 2020). Of course, this unexplained variation also reflects measurement error inherent in molecular characterization of soil microbial communities, including variation in DNA extraction efficiency, PCR biases, and sequencing error. We found significant overlaps between spatial factors, crop rotation, and soil chemistry in explaining microbial variances. Disentangling the autocorrelation between environmental and spatial factors remains a major methodological challenge in biogeographic studies, which requires careful design of a hierarchical sampling scheme (Baker et al. 2009).

(3) *Do edaphic and management factors have consistent or region‐specific effects on the growing‐season soil microbial abundance and diversity?* We found that growing‐season soil microbial properties in the Upper Midwest potato‐growing regions were strongly influenced by soil pH, SOM, rotation length and diversity, and pre‐plant fumigation. These relationships were region‐dependent, which is consistent with many past studies (Franklin and Mills 2003; Tedersoo et al. 2014; Yang et al. 2017; Liu et al. 2018; Ni et al. 2018; Bastida et al. 2021).

The predominant role of soil pH in shaping soil microbial communities has been demonstrated by many studies across varying ecosystems (Bahram et al. 2018; Wang et al. 2019; Tedersoo et al. 2020). Consistent with these studies, we found that pH was an overarching edaphic factor dominating soil bacterial and fungal taxonomic diversity. However, the impact of pH on microbial taxonomic diversity varied among regions, which may be attributed to the differences in pH ranges across these regions. Large scale across‐ecosystem surveys often show a unimodal relationship between soil pH and bacterial taxonomic diversity: bacterial diversity first increases as soil pH increases from 3.5 to 7, then declines as pH increases from 7 to 9 (Fierer and Jackson 2006). A similar relationship has also been reported for fungal taxonomic diversity in forest soils where fungal diversity increased from pH 3 to 5.5, and then decreased from 5.5 to 7 (Tedersoo et al. 2020). In our study, the soil pH in the sand and loam regions ranged from 4.5 to 8, which is within the range where bacterial diversity tends to increase and fungal diversity decreases with pH. In contrast, the peat region had a narrow pH range (6.5–8), where both bacterial and fungal taxonomic diversity appeared to decrease as pH increased. In general, our result shows one unimodal relationship between pH and microbial taxonomic diversity across all regions.

The impact of pH on microbial abundance also varied among regions, with differences occurring between the sand and peat/loam regions. In peat and loam regions, increasing soil pH was associated with increased bacterial and fungal abundance; this pattern was not found in the sand region. A positive impact of soil pH on soil microbial biomass has been reported in other studies. For example, Tian et al. (2008) observed an increase in soil microbial biomass as pH rose from 4.1 to 7.9 in a natural pH gradient in forest soils. Similar results have been found in soils collected from various agriculture sites with a wide range of pH values (Li et al. 2009; Wang et al. 2019). The varying relationships between pH and microbial biomass among regions could be attributed to other co‐limiting factors, such as SOM content. The sand region soils generally contained lower percentages of SOM compared to the loam and peat regions. This low amount of SOM can inhibit the impact of increasing pH on microbial biomass if SOM and pH co‐limit the growth of microbial biomass. Furthermore, a low amount of SOM may also weaken the indirect effect of pH on microbial growth through the path of SOM turnover (Shao et al. 2022). Decomposition of SOM is accelerated with increasing availability of macronutrients as soil pH increases from 4 to 7.5 (Barrow and Hartemink 2023). In addition, plants may allocate more carbon into the soil in higher pH environments, which further contributes to the increased availability of carbon resources for microbial growth (Malik et al. 2018; Meng et al. 2019). In sandy soils with low availability of SOM, this indirect pH impact on microbial growth might be minimal.

Soil organic matter content is suggested to be intrinsically linked to the diversity and biomass of soil microorganisms (Delgado‐Baquerizo et al. 2016; Siles et al. 2023; Yiallouris et al. 2024). We found that SOM content had a stronger impact on bacterial than fungal abundance and diversity. This may be due to the significantly lower fungal than bacterial abundance and diversity in these intensively managed agriculture soils. We also found that bacterial abundance and diversity responded to SOM differently between peat and the other two regions. Within the sand or loam regions, a unit increase of SOM was associated with a significant increase in the bacterial abundance and diversity (Figure 5). In comparison, in the peat region SOM did not correlate with bacterial abundance and diversity. Peat soils contained a wide range of SOM at higher percentages (12%–89% in peat, compared to a range of 1%–4.1% in loam and 0.4%–17% in sands), as well as a higher native bacterial abundance and diversity, likely a result of the high SOM content. This suggests that the effect of SOM on bacterial abundance may have saturated in the peat region as access to SOM becomes non‐limiting to bacterial growth above a certain threshold. At the lower levels of SOM present in sandy and loam soils, an increase in SOM leads to an increase in bacterial abundance and diversity because bacterial growth might still be limited by SOM. Additionally, within the peat region, increasing SOM is associated with decreasing pH (Figure S3), which could potentially counteract the positive effect of SOM on bacterial growth.

Increasing rotation length and diversity benefit microbial biomass and diversity. Crop rotation is generally thought to improve microbial properties and ecosystem services through increasing soil aggregate stability and water availability (Larkin et al. 2021). Empirical studies typically show varying results in systems with different rotation crops. Increasing the diversity of rotation crops increased microbial biomass (Alhameid et al. 2019) and diversity (D'Acunto et al. 2018) in corn fields. Qin et al. (2022) found rotation fields showed lower bacterial diversity and higher fungal diversity than continuous potato cropping systems. Global synthesis shows crop rotation can increase microbial biomass and diversity, but the effect is mediated by other factors, including the type of crop, climate, and tillage, etc. (McDaniel et al. 2014; Venter et al. 2016; Liu et al. 2023). Our results showed that in the potato growing systems of the Upper Midwest, rotation length was positively related to bacterial biomass while rotation diversity was positively related to bacterial and fungal diversity. Rotation length may affect microbial biomass through the frequency of soil disturbance and the amount of plant residue left in the soil. Potato production requires frequent soil disturbance (tillage prior to planting, planting seed tubers, hilling, and harvest of the belowground crop), so the longer period between potato production years almost inevitably involves less disturbance, no matter what other crops are grown. Accumulation of plant residue in these soils could benefit microbial biomass accumulation (Carter and Sanderson 2001; Nelson et al. 2009). Microbial diversity might be more sensitive to the variety of plant species and their associated litter qualities, making it particularly sensitive to crop diversification (Ai et al. 2018; Morugán‐Coronado et al. 2022). These rotation impacts were most pronounced in the loam region. In the sand region, rotation length had a stronger influence on microbial properties than rotation diversity (Figure 3), suggesting that management practices with minimal soil disturbances may be more important in regulating organic matter accumulation and soil microbial properties in these low organic soils. In the peat region, bacterial properties were less responsive to both rotation length and diversity, possibly due to the inherently high bacterial abundance in these soils.

Fumigation is a widely adopted practice in potato production with the goal of yield promotion and disease prevention. As a broad‐spectrum fungicide, fumigation has a potential negative impact on soil microbiota. This multi‐state survey found that fall fumigation primarily affected diversity rather than the abundance of soil bacterial communities in the following growing season. This suggests that fumigation likely altered the community composition by suppressing certain taxa while enhancing others. Fumigation only decreased bacterial diversity in the loamy soils and had minimal impact in the sandy soil, suggesting that organic matter content might be an important factor mediating fumigation impact on soil microbiota (Shan et al. 2024).

Our results suggest that soil microbial taxonomic diversity is decoupled from functional diversity. Despite the contribution of pH, SOM, rotation, and fumigation to microbial abundance and taxonomic diversity, these factors had little effect on functional diversity. For instance, soil organic matter, which generally increased microbial abundance and taxonomic diversity, did not affect functional diversity. This pattern suggests a high degree of functional redundancy in organic matter decomposition, as increased organic substrate availability may lead to the prefoliation of different taxonomic groups that utilize the same substrates (Maron et al. 2018). Similarly, taxonomic diversity did not translate into corresponding patterns in functional diversity. For instance, despite low microbial abundance and taxonomic diversity in low‐organic, coarse‐textured sandy soils, functional diversity remained high. This pattern may reflect greater functional specialization and narrower niche breadth under resource‐limited conditions. In response to limited nutrient and energy availability, microorganisms can adjust their metabolic traits and adopt distinct ecological niches in a more competitive environment, leading to high functional diversity despite low taxonomic diversity (Peng et al. 2025). These findings support the widely observed decoupling of taxonomic and functional diversities in response to environmental conditions in current literature (Souza et al. 2016; Fenoy et al. 2022; Wang et al. 2026). Nevertheless, these findings should be interpreted with caution because functional predictions based on reference databases are limited by our incomplete understanding of microbial gene and gene expression, particularly in fungal communities (Douglas et al. 2019; Ramond et al. 2025).

## 5 Conclusions

In conclusion, our three growing regions harboured distinct microbial communities that responded to soil chemical gradients and farm management practices in often unique ways. In our peat region, communities were characterized by high abundance and broad scale taxonomic diversity of bacteria, with correspondingly lower abundance and diversity of fungi. In contrast, our sandy soils, where the bulk of commercial potato production occurs, had low abundance of both bacteria and fungi and lower bacterial taxonomic diversity at a broad taxonomic scale, with loamy soils intermediate between these extremes. However, these regions also differed in how responsive the soil communities were to chemical properties and management practices. Our sandy soils represented an oligotrophic, resource‐limited environment supporting bacterial communities that responded to even slight increases in SOM but were constrained such that the communities showed minimal responses to management practices like rotation diversity or fumigation. On the other hand, the loamy soil was more responsive to rotation and fumigation, perhaps because the higher levels of organic matter provided enough of a resource base for bacterial populations to respond positively to reduced disturbance or higher plant diversity. Finally, in the very rich peat soils, bacterial abundance and (broad‐scale) taxonomic diversity were high in all samples and thus non‐responsive to management for the opposite reason.

Soils are complex systems that reflect chemical, physical, and biological properties that all have the potential to respond in unique ways to historical factors on scales from millennia to months. As such, attempts to define a proscribed set of metrics representing “healthy soils” may be difficult. Instead, what constitutes “healthy” soils may be specific to spatial regions, soil types, or production systems. Here we investigated how microbial communities in potato production fields in the upper Midwest varied across spatial, temporal, chemical, and management gradients. Importantly, we found that different aspects of microbial communities (fungi vs bacteria, abundance vs diversity) responded to different soil properties and management factors, highlighting the difficulty in defining a single set of practices to promote “healthy” microbial communities even in this one production system. Moreover, most of the relationships between soil chemistry, management, and soil microbial communities were regionally dependent, differing in strength and even direction across our three soil type regions. This further highlights the need to develop region‐specific recommendations for soil health promoting practices; “one‐size‐fits‐all” approaches to soil management are likely to result in ineffective or even counterproductive outcomes.

## Author Contributions

**Shan Shan:** data curation, writing – review and editing, writing – original draft, formal analysis, methodology. **Isabelle George:** data curation, methodology. **Linda L. Kinkel:** conceptualization, funding acquisition, methodology. **Richard A. Lankau:** conceptualization, funding acquisition, methodology, writing – review and editing.

## Funding

This work was supported by the U.S. Department of Agriculture (SCMP1701).

## Conflicts of Interest

The authors declare no conflicts of interest.

## Supporting information


**Table S1:** Variance partitioning showing the effect of season (Se), spatial location (Sp), soil chemistry (Ch), and management history (Ma) on microbial taxonomic diversity and composition at various taxonomic scales. Values show the percentage of explained variance by each factor or joined effects by multiple factors, corresponding to the Upset plots in Figure 3.
**Table S2:** Variance partitioning showing the effect of season (Se), spatial location (Sp), soil chemistry (Ch), and management history (Ma) on microbial abundance, functional diversity and functional composition. Values show the percentage of explained variance by each factor or joined effects by multiple factors, corresponding to the Upset plots in Figure 4.
**Figure S1:** Histograms showing the distribution of 16S and ITS reads per sample for the 1270 soil samples. Sample depth of coverage. Histogram of the mean sequencing read depth per sample for 641 samples. 1.7% of the samples (22 samples) have number of 16S reads less than 1800. 3.1% of the samples (40 samples) have number of ITS reads less than 700.
**Figure S2:** Principal coordinate analysis (PCoA) of bacterial and fungal taxonomic and functional compositions based on Bray–Curtis dissimilarity. Functional profile was inferred from KO abundance for bacteria and EC abundance for fungi. The effect of season was detected by permutation multivariate analysis of variance. ***, *p* < 0.001.
**Figure S3:** Negative correlation between pH and soil organic matter content in soils collected from the peat region.

## Data Availability

The 16S and ITS amplicon sequencing data are publicly available at the NCBI Short Read Archive under BioProject PRJNA1135141. Other data are available from the USDA Ag Data Commons: https://doi.org/10.15482/USDA.ADC/29944829.
