# Supplementary material for: Regionally Divergent Relationships Between Microbial Communities and Environmental Gradients in Potato Production Systems
Source: Environ Microbiol Rep. 2026 Oct 20;18(5):e70437. doi: 10.1111/1758-2229.70437 (PMC13681253; doi:10.1111/1758-2229.70437)
Supplement: Supplementary file 1 [file EMI4-18-e70437-s001.docx]

Supplemental materials for “Regionally divergent relationships between microbial communities and environmental gradients in potato production systems”

Table S1. Variance partitioning showing the effect of season (Se), spatial location (Sp), soil chemistry (Ch), and management history (Ma) on microbial taxonomic diversity and composition at various taxonomic scales. Values show the percentage of explained variance by each factor or joined effects by multiple factors, corresponding to the Upset plots in Fig. 3.

| *Taxonomic diversity* | | | |  | *Taxonomic composition* | | | |
| --- | --- | --- | --- | --- | --- | --- | --- | --- |
| Bacteria | | Fungi | |  | Bacteria | | Fungi | |
| *Class* |  |  |  |  |  |  |  |  |
| [a] Se only | 1.2 | [a] Se only | 2.7 |  | [a] Se only | 1.2 | [a] Se only | 3.9 |
| [b] Sp only | 19.3 | [b] Sp only | 12.9 |  | [b] Sp only | 18.0 | [b] Sp only | 19.3 |
| [c] Ch only | 0.8 | [c] Ch only | 0.4 |  | [c] Ch only | 1.6 | [c] Ch only | 0.2 |
| [f] Se+Ch | 30.9 | [d] Ma only | 0.2 |  | [d] Ma only | <0.1 | [d] Ma only | 0.3 |
| [i] Sp+Ma | 1.8 | [f] Se+Ch | 19.4 |  | [f] Se+Ch | 27.2 | [f] Se+Ch | 10.3 |
| [m] Se+Ch+Ma | 9.7 | [i] Sp+Ma | 0.6 |  | [i] Sp+Ma | 0.9 | [i] Sp+Ma | 0.5 |
| [o] Se+Sp+Ch+Ma | <0.1 | [m] Se+Ch+Ma | 12.7 |  | [m] Se+Ch+Ma | 14.5 | [l] Se+Sp+Ma | <0.1 |
|  |  |  |  |  | [n] Sp+Ch+Ma | <0.1 | [m] Se+Ch+Ma | 4.8 |
|  |  |  |  |  |  |  | [n] Sp+Ch+Ma | <0.1 |
| *Order* |  |  |  |  |  |  |  |  |
| [a] Se only | 0.1 | [a] Se only | 3.9 |  | [a] Se only | 1.6 | [a] Se only | 3.7 |
| [b] Sp only | 28.6 | [b] Sp only | 17.3 |  | [b] Sp only | 18.5 | [b] Sp only | 22.6 |
| [c] Ch only | 0.2 | [c] Ch only | 0.2 |  | [c] Ch only | 1.6 | [c] Ch only | 0.2 |
| [d] Ma only | 0.0 | [d] Ma only | 0.2 |  | [d] Ma only | <0.1 | [d] Ma only | 0.3 |
| [f] Se+Ch | 6.3 | [f] Se+Ch | 8.4 |  | [f] Se+Ch | 27.0 | [f] Se+Ch | 8.6 |
| [i] Sp+Ma | 2.1 | [i] Sp+Ma | 0.7 |  | [i] Sp+Ma | 0.8 | [i] Sp+Ma | 0.8 |
| [o] Se+Sp+Ch+Ma | <0.1 | [m] Se+Ch+Ma | 8.2 |  | [m] Se+Ch+Ma | 14.7 | [l] Se+Sp+Ma | 0.1 |
|  |  |  |  |  | [n] Sp+Ch+Ma | <0.1 | [m] Se+Ch+Ma | 3.5 |
|  |  |  |  |  |  |  | [o] Se+Sp+Ch+Ma | 0.1 |
| *Family* |  |  |  |  |  |  |  |  |
| [a] Se only | 0.7 | [a] Se only | 7.6 |  | [a] Se only | 1.6 | [a] Se only | 3.0 |
| [b] Sp only | 19.5 | [b] Sp only | 26.6 |  | [b] Sp only | 18.7 | [b] Sp only | 23.9 |
| [c] Ch only | 0.6 | [d] Ma only | 0.1 |  | [c] Ch only | 1.6 | [c] Ch only | 0.3 |
| [d] Ma only | 0.1 | [f] Se+Ch | 4.0 |  | [d] Ma only | <0.1 | [d] Ma only | 0.2 |
| [f] Se+Ch | 14.0 | [i] Sp+Ma | 0.7 |  | [f] Se+Ch | 27.5 | [f] Se+Ch | 9.3 |
| [i] Sp+Ma | 2.1 | [m] Se+Ch+Ma | 4.0 |  | [i] Sp+Ma | 0.8 | [i] Sp+Ma | 1.1 |
| [o] Se+Sp+Ch+Ma | 0.0 |  |  |  | [m] Se+Ch+Ma | 15.0 | [l] Se+Sp+Ma | 0.1 |
|  |  |  |  |  | [n] Sp+Ch+Ma | <0.1 | [m] Se+Ch+Ma | 4.1 |
|  |  |  |  |  |  |  | [o] Se+Sp+Ch+Ma | 0.1 |
| *Genus* |  |  |  |  |  |  |  |  |
| [a] Se only | 0.1 | [a] Se only | 5.3 |  | [a] Se only | 1.7 | [a] Se only | 2.8 |
| [b] Sp only | 17.8 | [b] Sp only | 26.1 |  | [b] Sp only | 19.3 | [b] Sp only | 24.9 |
| [d] Ma only | 0.3 | [f] Se+Ch | 2.3 |  | [c] Ch only | 1.7 | [c] Ch only | 0.3 |
| [f] Se+Ch | 7.1 | [i] Sp+Ma | 1.4 |  | [d] Ma only | <0.1 | [d] Ma only | 0.1 |
| [i] Sp+Ma | 1.0 | [m] Se+Ch+Ma | 0.7 |  | [f] Se+Ch | 26.5 | [f] Se+Ch | 9.1 |
| [j] Ch+Ma | <0.1 |  |  |  | [i] Sp+Ma | 0.8 | [i] Sp+Ma | 1.3 |
| [m] Se+Ch+Ma | 0.3 |  |  |  | [m] Se+Ch+Ma | 13.8 | [k] Se+Sp+Ch | <0.1 |
|  |  |  |  |  | [n] Sp+Ch+Ma | <0.1 | [l] Se+Sp+Ma | 0.1 |
|  |  |  |  |  |  |  | [m] Se+Ch+Ma | 3.5 |
|  |  |  |  |  |  |  | [o] Se+Sp+Ch+Ma | 0.1 |
| *ASV* |  |  |  |  |  |  |  |  |
| [b] Sp only | 19.3 | [a] Se only | 2.9 |  | [a] Se only | 0.7 | [a] Se only | 2.0 |
| [d] Ma only | 0.1 | [b] Sp only | 21.0 |  | [b] Sp only | 24.8 | [b] Sp only | 27.3 |
| [f] Se+Ch | 2.2 | [c] Ch only | <0.1 |  | [c] Ch only | 1.0 | [c] Ch only | 0.3 |
| [g] Se+Ma | <0.1 | [f] Se+Ch | 5.6 |  | [d] Ma only | <0.1 | [d] Ma only | 0.1 |
| [i] Sp+Ma | 1.8 | [i] Sp+Ma | 1.0 |  | [f] Se+Ch | 15.7 | [f] Se+Ch | 9.7 |
| [m] Se+Ch+Ma | <0.1 | [j] Ch+Ma | 0.0 |  | [i] Sp+Ma | 1.1 | [i] Sp+Ma | 1.5 |
| [o] Se+Sp+Ch+Ma | <0.1 | [m] Se+Ch+Ma | 0.7 |  | [m] Se+Ch+Ma | 7.0 | [k] Se+Sp+Ch | <0.1 |
|  |  |  |  |  | [n] Sp+Ch+Ma | <0.1 | [l] Se+Sp+Ma | 0.1 |
|  |  |  |  |  |  |  | [m] Se+Ch+Ma | 3.1 |
|  |  |  |  |  |  |  | [o] Se+Sp+Ch+Ma | 0.1 |

Table S2. Variance partitioning showing the effect of season (Se), spatial location (Sp), soil chemistry (Ch), and management history (Ma) on microbial abundance, functional diversity and functional composition. Values show the percentage of explained variance by each factor or joined effects by multiple factors, corresponding to the Upset plots in Fig. 4.

| *Abundance* | | | | | |
| --- | --- | --- | --- | --- | --- |
| Bacteria | |  |  | Fungi | |
| [a] Se only | 0.1 |  |  | [b] Sp only | 22.4 |
| [b] Sp only | 12.1 |  |  | [c] Ch only | 0.1 |
| [c] Ch only | 0.5 |  |  | [f] Se+Ch | 9.4 |
| [d] Ma only | 0.0 |  |  | [g] Se+Ma | <0.1 |
| [f] Se+Ch | 37.2 |  |  | [h] Sp+Ch | <0.1 |
| [i] Sp+Ma | 2.3 |  |  | [i] Sp+Ma | 1.4 |
| [k] Se+Sp+Ch | <0.1 |  |  |  |  |
| [l] Se+Sp+Ma | <0.1 |  |  |  |  |
| [m] Se+Ch+Ma | 21.1 |  |  |  |  |
| *Functional diversity* | | | | | |
| Bacteria | | | | Fungi | |
| *Pathway* |  | *KO* |  | *EC* | |
| [a] Se only | 0.1 | [a] Se only | <0.1 | [a] Se only | 1.1 |
| [b] Sp only | 18.2 | [b] Sp only | 18.9 | [b] Sp only | 22.0 |
| [c] Ch only | 0.5 | [c] Ch only | 0.3 | [d] Ma only | 0.1 |
| [e] Se+Sp | 0.1 | [e] Se+Sp | 0.1 | [f] Se+Ch | 6.2 |
| [f] Se+Ch | 22.0 | [f] Se+Ch | 22.4 | [h] Sp+Ch | <0.1 |
| [g] Se+Ma | <0.1 | [g] Se+Ma | <0.1 | [k] Se+Sp+Ch | <0.1 |
| [i] Sp+Ma | 0.4 | [h] Sp+Ch | <0.1 | [m] Se+Ch+Ma | 0.4 |
| [j] Ch+Ma | <0.1 | [i] Sp+Ma | 0.3 |  |  |
| [k] Se+Sp+Ch | <0.1 | [k] Se+Sp+Ch | <0.1 |  |  |
| [m] Se+Ch+Ma | 11.7 | [m] Se+Ch+Ma | 11.4 |  |  |
| *Functional composition* | | | | | |
| Bacteria | | | | Fungi | |
| *Pathway* | | *KO* | | *EC* | |
| [a] Se only | 1.2 | [a] Se only | 1.4 | [a] Se only | 2.5 |
| [b] Sp only | 21.0 | [b] Sp only | 21.3 | [b] Sp only | 21.6 |
| [c] Ch only | 1.2 | [c] Ch only | 1.3 | [c] Ch only | 0.1 |
| [d] Ma only | <0.1 | [d] Ma only | <0.1 | [d] Ma only | 0.1 |
| [f] Se+Ch | 22.3 | [f] Se+Ch | 21.3 | [f] Se+Ch | 7.8 |
| [i] Sp+Ma | 0.9 | [i] Sp+Ma | 0.8 | [i] Sp+Ma | 0.3 |
| [l] Se+Sp+Ma | <0.1 | [l] Se+Sp+Ma | 0.1 | [k] Se+Sp+Ch | <0.1 |
| [m] Se+Ch+Ma | 7.9 | [m] Se+Ch+Ma | 7.2 | [m] Se+Ch+Ma | 2.3 |
| [n] Sp+Ch+Ma | <0.1 | [n] Sp+Ch+Ma | <0.1 | [o] Se+Sp+Ch+Ma | <0.1 |
| [o] Se+Sp+Ch+Ma | 0.1 | [o] Se+Sp+Ch+Ma | 0.1 |  |  |

Fig. S1 Histograms showing the distribution of 16S and ITS reads per sample for the 1270 soil samples. Sample depth of coverage. Histogram of the mean sequencing read depth per sample for 641 samples. 1.7 % of the samples (22 samples) have number of 16S reads less than 1800. 3.1% of the samples (40 samples) have number of ITS reads less than 700.

Fig. S2 Principal coordinate analysis (PCoA) of bacterial and fungal taxonomic and functional compositions based on Bray-Curtis dissimilarity. Functional profile was inferred from KO abundance for bacteria and EC abundance for fungi. The effect of season was detected by permutation multivariate analysis of variance. ***, P<0.001.

Fig. S3 Negative correlation between pH and soil organic matter content in soils collected from the peat region.
